# Lithium-induced Cardiotoxicity: A Rare Clinical Entity

**DOI:** 10.7759/cureus.7286

**Published:** 2020-03-16

**Authors:** Sudeep Acharya, Abdul Hasan Siddiqui, Shamsuddin Anwar, Saad Habib, Shamsuddin Anwar

**Affiliations:** 1 Internal Medicine, Staten Island University Hospital, Staten Island, USA; 2 Pulmonary and Critical Care Medicine, University of Illinois Urbana Champaign, Champaign, USA; 3 Internal Medicine, Staten Island University Hospital, Staten Island , USA

**Keywords:** arrhythmia, lithium, cardiotoxicity, pulmonary critical care

## Abstract

Lithium has been used effectively used in the management of mood disorders, such as bipolar disease, acute mania, and hypomania. As the therapeutic index is very narrow for lithium, it is important to monitor lithium levels periodically to avoid toxic effects. Common toxic effects include diarrhea, tremor, muscle weakness, ataxia, and myoclonus. Severe toxicity can present with seizures, coma, and death. Cardiotoxicity secondary to lithium is rarely reported in the medical literature and can range from dysrhythmias and cardiomyopathies to myocardial infarction. We describe an interesting case report of cardiac toxicity secondary to lithium in a bipolar patient managed conservatively in an intensive care setting.

## Introduction

Lithium is a medication that is used for several mood disorders [1-2]. It has been widely used for bipolar disorders effectively. Several side effects have been reported secondary to lithium which mandates its levels to be monitored cautiously. Generally, the side effects, although reversible, are related to the neurological and gastrointestinal tract. However, the cardiac side effects are not unheard of in the medical literature [3]. We report an interesting case of cardiotoxicity induced by lithium in a patient and the successful treatment with conservative measures. 

## Case presentation

A 57-year-old male with a past medical history of hypothyroidism, borderline mental retardation, seizure disorder, and bipolar disease on chronic lithium therapy was sent from a group home for evaluation of a change in mental status. His other medications included clonazepam, carbamazepine, lacosamide, valproic acid, levothyroxine, and atorvastatin. The patient was obtunded on presentation, so the medical history was obtained from the accompanying aid from the group home. The patient initially developed progressive generalized weakness since a recent (one week) increase of his lithium dosage from 300 mg to 450 mg twice a day. Over the course of time, he became progressively non-responsive, lethargic, and was having difficulty clearing his secretions in the upper airway tract. There were no reports of any fever, cough, chest pain, change in bowel habits, urinary complaints, abdominal discomfort, or traumatic injury.

On presentation to the emergency room, the patient was completely obtunded. Initial vital signs were significant for tachypnea and bradycardia; his blood pressure was stable and the temperature was normal. His physical examination was significant for inspiratory stridor with bibasilar crackles. The other review of systems were within normal limits; neurological evaluation was limited as the patient was unable to follow commands.

The presenting laboratory workup revealed a white cell count of 15,000/ml (normal: 4,000 - 11,000 cells/ml), mild elevation of aspartate aminotransferase of 59 units/liter (normal: 5 - 40 units/liter), and lithium level of 1.8 mmol/L (normal: 0.6 - 1.2 mmol/L) as the only significant findings. The rest of the blood profile and electrolytes were within normal ranges, including cardiac enzymes. 

**Figure 1:**
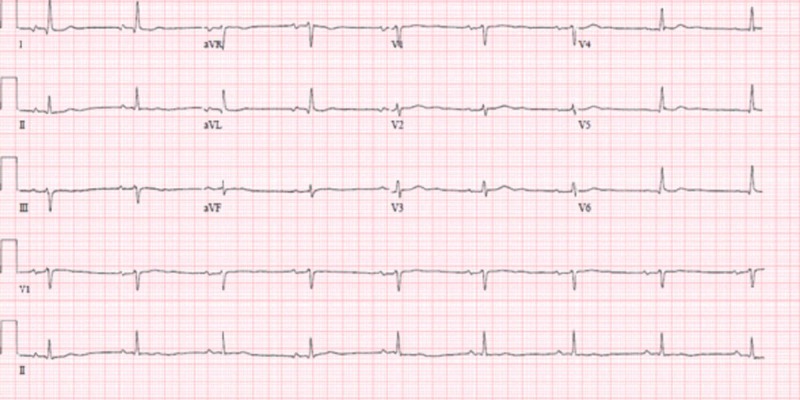
First electrocardiogram showing sinus bradycardia with a first-degree atrioventricular (AV) block

As seen in Figure 1, the electrocardiogram demonstrated sinus bradycardia with a heart rate of 54 with first-degree atrioventricular block and QRS wave and QTc within normal limits. The blood gas performed on admission demonstrated hypoxia without hypercapnia. With the mentation being the most important concern, along with respiratory distress and stridor, the patient was intubated for airway protection, initiated on sedation, and prophylactic antibiotics for suspected aspiration pneumonitis were started.

The patient was further investigated with computed tomography (CT) of the head to rule out an acute intracranial pathology, such as stroke vs. hemorrhage. A spot electroencephalogram was also performed to capture any seizure-like activity, which was also negative.

**Figure 2:**
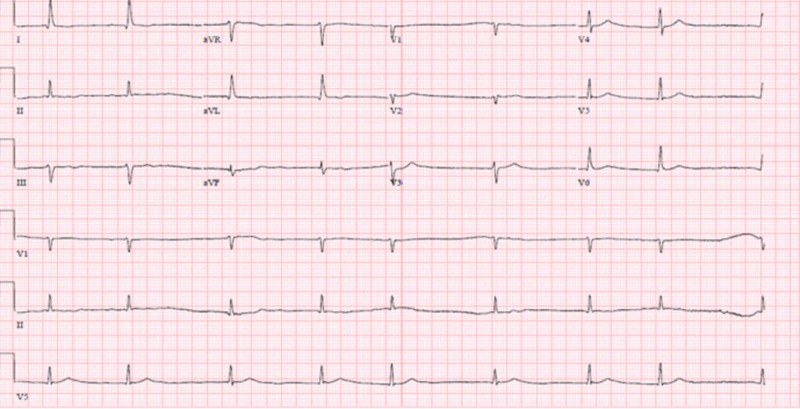
Second electrocardiogram showing atrial fibrillation with a slow ventricular response

**Figure 3:**
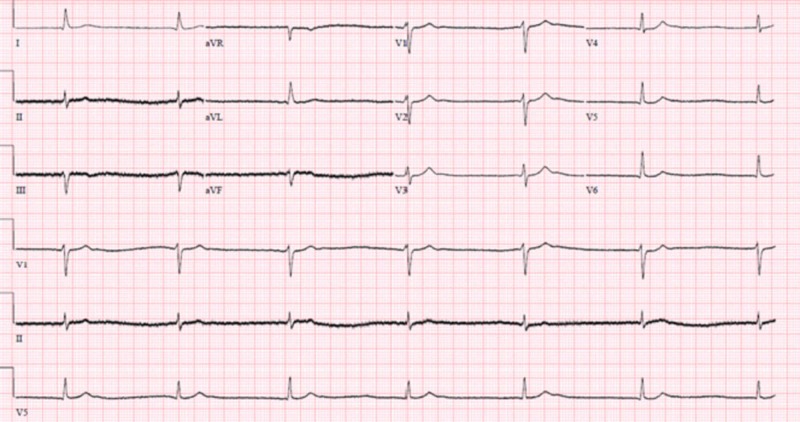
Third electrocardiogram showing junctional bradycardia

Over the course of 24 hours, the patient started to become profoundly bradycardic with the initiation of intravenous (IV) pushes of atropine, progressing to requiring transcutaneous pacing. The follow-up EKG as shown in Figures 2-3 progressed from atrial fibrillation with a slow ventricular response to junctional bradycardia with sinus pauses that lasted for a maximum time period of 10 seconds on telemonitoring. As the patient was becoming hypotensive and bradycardic, he was initiated on vasopressor support with dopamine and norepinephrine drip.

The primary diagnosis of cardiotoxicity secondary to an elevated lithium level was established, and the patient was also evaluated by cardiology and nephrology for a further recommendation in the plan of care. The consideration for initiating hemodialysis and a temporary pacemaker was discussed with the primary critical care team. As the lithium levels had already started to trend down with conservation measures, the consensus was established to hold off with dialysis and continue to monitor. The patient had already received a total of two liters of IV fluids in the first 24-hour time period of admission and the lithium levels had trended down to 1.3 mmol/L.

Eventually, the patient regained normal sinus rhythm, was extubated, and based on psychiatric recommendations, was placed on clonazepam and quetiapine with adjusted doses instead of lithium. He was ultimately discharged to the group home with normal kidney function and a normal heart rate.

## Discussion

Lithium has been effectively used for the treatment of mood disorders, like acute mania, hypomania, and bipolar disorders, for a long time [1-2]. The usual initial dose of lithium is 300 mg two to three times daily, which is gradually titrated upon a weekly basis according to an individual’s tolerance, body mass index, and response to the medication [4]. The goal of the dose titration is to approach a therapeutic index of 0.6 - 1.2 mmol/L with a steady regimen [5].

The narrow therapeutic index for lithium mandates close monitoring. Toxic effects are usually recognized with blood levels > 1.5 mmol/L, and severe toxicity is considered with levels > 2.5 mmol/L. As lithium has good oral bioavailability, soon after oral ingestion the peak blood levels may be reached within one to two hours. It has a half-life of approximately 18 hours in adults and 36 hours in the elderly [6].

Mild intoxication results in a variety of symptoms, such as diarrhea, vomiting, coarse hand tremors, and muscle weakness. Moderate intoxication can present with ataxia, myoclonus, and confusion. Severe intoxication causes seizures, impaired consciousness, coma, and death. While neurological and renal abnormalities are commonly reported, there is a scarcity of literature related to cardiac toxicity and the management of lithium-induced cardiotoxicity. Our case highlights the severe form of cardiac toxicity in a patient with high lithium levels (1.8 mmol/L) with no obvious renal function abnormality. The cardiac toxicities reported in the literature secondary to lithium have mostly been managed by either hemodialysis and temporary or permanent pacemaker placement. Our clinical case is unique in the sense that the patient was managed conservatively without aggressive measures, such as urgent hemodialysis and permanent pacemaker placement.

Cardiac effects attributed to lithium toxicity infrequently have been reported in the medical literature [7]. Although the mechanism of action for lithium has not been completely defined, the drug is suggested to exert its effect on sodium transporter channels in neurons, thereby altering the metabolism and storage of catecholamines. It is implied that lithium interferes with sodium/calcium exchanger and sodium/potassium pump resulting in disturbances of cardiac cell membrane physiology. These changes can cause hypercalcemia and hypokalemia, thereby interfering in electrical impulse propagation and depolarization manifested on electrocardiograms [8-9]. A wide range of these cardiac effects has been discussed in the literature that includes junctional rhythm, atrial fibrillation, ST-segment and T-wave changes, atrioventricular blocks, and sinus node arrhythmias [10-13]. It is of note that the aforementioned electrocardiographic changes are usually demonstrated in patients who may have chronically elevated lithium levels, especially > 1.5 mmol/L, but such arrhythmias can be precipitated with changes in lithium levels in a short time interval, as seen in our clinical case [14].

Although supratherapeutic levels of lithium are usually attributed to cardiac toxicity, the electrocardiographic changes have also been reported in patients who have been maintained properly in the therapeutic range [15-16]. This suggests that several other risk factors may also be playing a role in lithium-induced cardiotoxicity. It is recommended to closely monitor hydration status, renal function, and salt balance in individuals on lithium. Caution should also be taken in individuals already on other mood stabilizers and anti-seizure medications as such medications can interact with the lithium effect on ion channels [17].

The current management plan for lithium toxicity includes supportive care with hydration, hemodialysis, and cardiac pacing (temporary or permanent). Usually, the cardiotoxicity is reversible, and individuals revert to baseline as soon as lithium levels trend to normalize [18].

## Conclusions

Cardiotoxicity is an uncommon side effect of lithium. It can present with variety arrhythmias, including sinoatrial arrhythmias, atrial fibrillation, and nonspecific ST-segment changes, that mandate further evaluation of the individual, especially for electrolytes, renal function, and lithium levels. The main treatment for cardiotoxicity is the removal of lithium from the system via intravenous hydration, hemodialysis, and the use of supportive care, such as pacing.
