# Correction: Lithium-induced Cardiotoxicity: A Rare Clinical Entity

**DOI:** 10.7759/cureus.c33

**Published:** 2020-08-06

**Authors:** Sudeep Acharya, Abdul Hasan Siddiqui, Shamsuddin Anwar, Saad Habib, Rabih Maroun

**Affiliations:** 1 Internal Medicine, Staten Island University Hospital, Northwell Health, Staten Island, USA; 2 Pulmonary and Critical Care Medicine, University of Illinois at Urbana-Champaign, Champaign, USA; 3 Internal Medicine, Staten Island University Hospital, Staten Island, USA; 4 Internal Medicine, Staten Island University Hospital, Staten Island , USA

Due to a technical error, the name of the third author, Shamsuddin Anwar, was incorrectly repeated as the fifth author. The fifth author should have been listed as Rabih Maroun. The authors have been updated accordingly. Cureus sincerely regrets the error.

